# Transdiagnostic associations across communication, cognitive, and behavioural problems in a developmentally at-risk population: a network approach

**DOI:** 10.1186/s12887-019-1818-7

**Published:** 2019-11-21

**Authors:** Silvana Mareva, the CALM team, Joni Holmes

**Affiliations:** Medical Research Council Cognition and Brain Sciences Unit, University of Cambridge, 15 Chaucer Road, Cambridge, CB2 7EF UK

**Keywords:** ADHD, Language, Executive function, Learning difficulties, Behaviour problems, Network analysis

## Abstract

**Background:**

Communication, behavioural, and executive function problems often co-occur in childhood. Previous attempts to identify the origins of these comorbidities have typically relied on comparisons of different deficit groups and/or latent variable models. Here we apply a network approach to a heterogeneous sample of struggling learners to conceptualise these comorbidities as a dynamic system of interacting difficulties.

**Methods:**

714 children struggling with attention, learning, and/or memory were included. The sample consisted of children with both diagnosed (41%) and undiagnosed difficulties. The conditional independence network of parent ratings of everyday behaviour, cognition, and communication was estimated.

**Results:**

A clustering coefficient identified four interconnected areas of difficulty: (1) structural language and learning; (2) pragmatics and peer relationships; (3) behavioural and emotional problems; and (4) cognitive skills. Emotional and behavioural symptoms shared multiple direct connections with pragmatic abilities and cognitive problems, but not with structural language skills or learning problems. Poor structural language and cognitive skills were associated with learning problems. Centrality indices highlighted working memory and language coherence as symptoms bridging different problem areas.

**Conclusion:**

The network model identified four areas of difficulty and potential bridging symptoms. Although the current analytic framework does not provide causal evidence, it is possible that bridging symptoms may be the origins of comorbidities observed on a dimensional level; problems in these areas may cascade and activate problems in other areas of the network. The potential value of applying a dynamic systems network approach to symptoms of developmental disorders is discussed.

## Background

Behavioural difficulties, poor communication skills, and everyday cognitive problems are common in children, and they often co-occur [1–5]. Each set of symptoms is commonly associated with a specific developmental disorder, and as a consequence is typically studied in groups of children in whom such problems are characteristic. For example, behavioural problems such as hyperactivity are typically studied in children with attention deficit hyperactivity disorder (ADHD), while communication difficulties such as poor speech are often studied in children with developmental language disorder (DLD). There are practical advantages to this categorical approach. It defines clear symptom-based criteria to inform practitioner decision-making about diagnoses and interventions. However, it fails to accommodate high rates of comorbidity across developmental disorders [6–10] and substantial heterogeneity within disorders [7, 11–13]. The aim of the current study was to move away from studying links between problems of behaviour, communication, and cognition in discrete groups. Instead, symptom associations are explored in a large heterogeneous sample of children with clinical and subclinical levels of difficulties.

Over the past decade, there has been a broad shift away from diagnosis-specific deficits toward identifying dimensions that cut across disorders conventionally considered to be distinct [14, 15]. This approach has been applied most widely to adult psychiatric conditions [16–18], but there is widespread recognition of its value for characterising developmental disorders in terms of underlying dimensions of symptoms [19–23]. One of the most common methods for understanding how symptoms are related uses latent variable models, a statistical method that groups variables based on shared variance to derive underlying dimensions of difficulties [24]. This technique has been used to identify dimensions of phonological and non-phonological skills in children with diagnosed DLD and dyslexia [25]; and separate latent constructs for inattention and hyperactivity in children with ADHD [26].

Network analysis offers an alternative approach to understanding symptom interrelations. Instead of identifying underlying dimensions, network models focus on symptom-level associations, allowing for the possibility that symptoms could interact to causally affect and activate one another [27, 28]. This framework is suited to conceptualising and evaluating the potential origins of comorbidities. For example, pragmatic difficulties characteristic of ASD, and hyperactivity problems common in ADHD often co-occur [7, 11, 29, 30]. From a network model perspective, this co-occurrence can be conceptualised as arising in a dynamic system in which symptoms traditionally linked with one developmental disorder might trigger and/or maintain symptoms commonly associated with a different disorder.

In the current study network science is used to understand comorbidities between problems with communication, executive functions, and behaviour. Symptoms of communication and behavioural problems co-occur in the general population and in children with developmental disorders such as ADHD [7, 11, 30, 31], ASD, DLD, and reading difficulties [20, 31–33]. Individuals with communication and/or behavioural problems, including those with diagnosed developmental disorders, often have additional deficits in executive functions (EFs) - the cognitive abilities regulating thoughts and behaviour [34]. EFs can be broadly construed as two distinct but related neurodevelopmental systems. Cool cognitive-based EFs encompass working memory, planning, and cognitive inhibition, and are associated with academic learning [32] and attention [33]; hot executive processes are associated with stronger affective valence and involve the regulation of emotional responses and social awareness [34].

It is not clear why communication, cognitive, and behavioural problems co-occur in childhood. These areas of difficulties likely share common environmental and genetic influences. Nonetheless, there are several possibilities about how they may interact dynamically. One is that deficits in cool EFs might underlie both behavioural and communication difficulties. Consistent with this, poor working memory has been shown to underpin problems in attention, behaviour, and structural language [33, 35], and also accounts for the relationship between hyperactivity and pragmatic communication problems [36]. Alternatively, associations between cool EFs and pragmatics could be mediated by difficulties with hot EFs. Difficulties in inhibitory control could lead to hyperactive-impulsive behaviour and consequently poor social communication skills. Children who regularly display difficult behaviour may have limited opportunities to socialise, and thereby fail to develop good communication skills. In line with this, hyperactive-impulsive behaviours have been shown to account for the relationship between inhibition and the ability to apply pragmatic rules in everyday situations [37]. Another possibility is that language deficits may directly and/or indirectly impact the ability to regulate cognition, behaviour, and communication [38, 39]. For example, difficulties with language may lead to peer rejection and academic difficulties, which in turn may trigger behavioural problems.

The current approach uses network modelling to estimate associations and conditional independence across symptoms. Clustering methods are then used to identify closely inter-connected symptoms that may, or may not, correspond to areas of difficulty commonly associated with categorical diagnoses such as ADHD or DLD. This novel approach allows us to identify where symptoms sit in the network (i.e., which symptoms sit together), and to quantify the importance of different symptoms within the network. This includes identifying bridging symptoms that have multiple strong links across problem domains/clusters. Symptoms linking problem domains may reflect causal processes and/or shared aetiological influences. The conceptual interpretation of bridging symptoms identified in cross-sectional networks is not straightforward but one possibility is that such symptoms may spread activation across the system and may be the origins of the comorbidities observed on a dimensional level [40]. All analyses are cross-sectional, exploratory, and data-driven. However, on the basis of co-morbid symptom presentations reported in the literature, it is predicted that multiple direct associations would emerge across pragmatic and behavioural difficulties (hot EFs), with additional connections between everyday cognitive abilities (cool EFs) and structural language skills.

## Methods

### Recruitment

The data reported are those collected between 2014 and 2018 at the Centre for Attention, Learning, and Memory (CALM). Children aged 5 to 18 years were referred to CALM by health and education practitioners for problems in the areas of attention, learning, and/or memory. Children were accepted into the study irrespective of diagnostic status, providing they met the following inclusion criteria: (1) native English speaker, (2) no uncorrected sensory impairments, and (3) no confirmed presence of genetic or neurological conditions. Full recruitment and testing procedures are described in the study protocol paper [41]. Children provided assent and parents/guardians provided informed written consent. Ethical approval was granted by the National Health Service (NHS) Health Research Authority NRES Committee East of England, REC approval reference 13/EE/0157.

### Assessments

The current analysis is based on the parent ratings of behaviour and communication described below. These checklists are routinely administered in health and educational settings in the United Kingdom to capture the child’s natural behaviour and communication in day-to-day situations.

### Brief rating inventory of executive function (BRIEF)

The Behaviour Rating Inventory of Executive Function (BRIEF) [42] is a parent checklist of everyday behaviours relating to: inhibition, shifting, emotional control, initiation, working memory, planning/organisation, organisation of materials, and monitoring*.*

### Conners-3

The Conners Parent Rating Short Form 3rd Edition [43] assesses ADHD-related difficulties across six subscales: inattention, hyperactivity/ impulsivity, learning problems, executive functioning, aggression, and peer relations. The executive functioning subscale was dropped as it was closely related to BRIEF scores (BRIEF Global Executive Score and Conners-3 EF – *rs* (687) = .67, *p* < .0001, two-tailed) and because the eight BRIEF subscales provide a more comprehensive assessment of EF.

### Children’s communication checklist (CCC-2)

The Children’s Communication Checklist (CCC-2) [44] contains ten subscales assessing communication. Speech, syntax, semantics, and coherence measure structural communication skills. Inappropriate initiation, stereotyped language, use of context, and nonverbal communication address pragmatic communication. The social relations and interests scales assess communication problems commonly observed in ASD*.*

### Participants

Parent ratings were available for 720 children. Outlier treatment was conducted as follows: three subscale scores were replaced with missing values due to falling ±3.5 standard deviations (SD) from the sample mean; six cases were detected as multivariate outliers based on Mahalanobis’ *D*^*2*^ and omitted from the analyses. Characteristics of the final sample of 714 children, including diagnostic status and referral route are available in Table 1.

**Table 1:** Demographics, diagnosis, and referral route

	Mean (SD)	Min-Max
Age (years)	9.4 (2.33)	5.17–18.58
	***N*** **male**	**% male**
Gender	480	67%
**Diagnosis**	**Total**	**%**
None	427	59
ADHD/ADD	187	26
Learning deficit	67	9
ASD	59	8
Other	33	5
Comorbidity	65	9
**Referrer**	**Total**	**%**
SENCo	395	55.32
Specialist Teacher	16	2.24
Educational Psychologist	7	0.98
Speech & Language Therapist	32	4.48
Clinical Psychologist	30	4.20
Child Psychiatrist	9	1.26
Paediatrician	183	25.63
ADHD nurse practitioner	30	4.2
Family worker locality team	10	1.4
Private Tutor	2	0.28

*ADHD* attention deficithyperactivity disorder, *ASD* autism spectrum disorder; *Learning deficit* Primary diagnoses of developmental language disorder, dyslexia, dyscalculia, or dysgraphia, *Other* Obsessive-compulsive disorder, depression, anxiety, *SENCo* special educational needs coordinator, *Comorbidity* presence of more than one diagnosis

### Missing data and overly negative scores

The number of participants with complete data for each scale is presented in Table 2. The network was estimated based on complete pairwise correlations to allow for the largest possible sample size (see Additional file 1: Figure S1 and S2 for alternative estimations). The analyses were conducted with and without ratings flagged as overly negative or inconsistent by the indices provided in each scale (See Additional file 1). The inclusion of these ratings did not alter the overall conclusions. The results presented here are for the full sample.

**Table 2:** Descriptive statistics, reliability, and percentage of children rated as experiencing clinical levels of difficulties

**Conners-3**	***N***	***M***	***SD***	***%*** ***T-score*** ***>= 60***	***Skew***	***Kurt***	***Min***	***Max***	***α***	***ω***
Inattention	702	11.68	3.44	90.48	-1.11	0.54	0	15	0.92	0.93
Hyperactivity/Impulsivity	704	10.96	5.63	75.77	-0.39	-1.11	0	18	0.95	0.96
Learning Problems	704	9.83	3.48	87.54	-0.42	-0.56	0	15	0.78	0.86
Aggression	701	3.55	3.99	49.58	1.21	0.55	0	15	0.93	0.94
Peer Relations	696	5.50	4.47	67.37	0.47	-0.97	0	15	0.93	0.93
**BRIEF**	***N***	***M***	***SD***	***%*** ***T-score >= 60***	***Skew***	***Kurt***	***Min***	***Max***	***α***	***ω***
Inhibition	707	21.51	6.23	60.5	-0.24	-1.21	10	30	0.97	0.98
Shifting	708	17.09	4.28	66.11	-0.27	-0.90	8	24	0.91	0.93
Emotional Control	707	22.12	5.59	61.9	-0.37	-0.93	10	30	0.95	0.97
Initiation	707	17.69	3.33	70.59	-0.37	-0.33	8	24	0.82	0.87
Working Memory	704	25.81	3.94	89.08	-1.14	0.89	12	30	0.93	0.95
Planning	698	29.06	4.97	85.15	-0.79	0.18	13	36	0.90	0.92
Organisation	708	14.62	3.22	56.58	-0.81	-0.26	6	18	0.92	0.95
Monitoring	706	18.98	3.53	68.21	-0.56	-0.36	9	24	0.86	0.92
**CCC-2**	**N**	**M**	**SD**	**%** **Scaled score** **<= 6**	**Skew**	**Kurt**	**Min**	**Max**	**α**	**ω**
Speech	702	5.18	5.03	62.75	1.10	0.55	0	21	0.90	0.94
Syntax	704	5.11	4.62	66.67	1.00	0.36	0	20	0.87	0.93
Semantics	702	8.16	4.74	78.15	0.37	-0.43	0	21	0.84	0.91
Coherence	705	8.57	5.13	77.17	0.21	-0.87	0	21	0.85	0.91
Inappropriate Initiation	702	10.79	5.71	68.21	-0.04	-1.07	0	21	0.88	0.91
Stereotyped Language	703	6.10	4.27	64.99	0.62	-0.25	0	20	0.80	0.88
Use of Context	704	9.58	5.48	77.87	0.05	-0.94	0	21	0.87	0.91
Nonverbal Communication	703	7.71	5.05	73.81	0.34	-0.81	0	21	0.84	0.90
Social Relations	703	6.61	4.94	70.87	0.49	-0.67	0	20	0.85	0.89
Interests	702	9.19	4.61	68.21	0.21	-0.62	0	21	0.80	0.87

If children’s age was outside the standardisation range, the closest age match was used. BRIEF = Behaviour Rating Inventory of Executive Function; CCC-2 = Children’s Communication Checklist 2. *M* = mean, *SD* = standard deviation, *α* = Cronbach’s alpha, *ω* = McDonald’s omega. For BRIEF and Conners-3, higher raw and *T*-scores values indicate presence of more difficulties. For CCC-2, lower raw scores indicate greater difficulties, whereas lower scaled scores indicate less difficulties

### Statistical analyses

Analyses were conducted in four steps: network estimation, network stability, community detection, and network inference based on centrality indices and predictability. All analysis were performed in *R* [45] using the packages qgraph [46], bootnet [47], mgm [48], igraph [49], and networktools [50].

### Network estimation

The regularised partial correlation network of the BRIEF, CCC-2, and Conner’s 3 subscales was estimated. Subscales had excellent to good internal consistency, suggesting that the items within each scale were capturing the same construct (Table 2). Therefore, the analysis focused on subscales’ interrelations to avoid potential bias introduced by the inclusion of multiple items assessing the same construct (see [51]). The distribution of the ratings on multiple subscales deviated from normality (see Table 2 for skew and kurtosis). For this reason, the network was estimated with nonparanormal transformation [52]. This transformation uses cumulative distributions to transform the observed variable to the distribution of the latent normally distributed variable (for details see [52, 53]). All raw scores were transformed such that higher values represented more difficulties and were standardised to the sample mean to put them on the same scale. Age was included in the estimation but omitted from plots and the calculation of the centrality indices. In the final network, each node represented a subscale rating and each edge corresponded to the regularised partial correlation coefficient across the two subscales, controlling for the influence of age and all other ratings. The network was estimated using the graphical variant of the least absolute shrinkage and selection operator (glasso) [54] to avoid including spurious edges. The best fitting model was selected based on the Extended Bayesian Information Criterion (EBIC) [55]. This method is reported to accurately retrieve the true network structure [56] and is described in detail elsewhere [47]. To ensure good specificity (including only edges that are truly present), the estimated elements of the inverse variance-covariance matrix were first thresholded using a theoretical bound [57]. This procedure reflects a strong assumption of sparsity and could result in loss of sensitivity to detect small edges that are truly present. In the current sample, the minimum absolute edge weight in the non-thresholded network was 0.0007; and 0.067 in the thresholded network. The implications of this analytical decision were tested by comparing the thresholded, non-thresholded, and unregularised solutions, all of which retained acceptable similarity (see Additional file 1 for details).

### Network stability

The robustness of the solution was scrutinised by calculating bootstrapped non-parametric 95% confidence intervals for all edges weights, together with the percentage of bootstrapped networks (*N* = 2000), in which the edge was estimated as different from zero (% non-zero) [47]. Additionally, as a coarse means to assess the possibility that the network structure may vary for different diagnostic groups represented in the sample, separate networks were estimated for children with no diagnosis (*N* = 389, excluding those with suspected ADHD) and for those with ADHD/ADHD under investigation (*N* = 227) and compared to the network generated for the whole sample. The ADHD group was chosen as it was the largest diagnosed subgroup within the sample. Both networks retained acceptable similarity with the network estimated from the full sample (adjacency matrices correlation ADHD/ADHD under investigation: *r* = .86; no diagnosis: *r* = .92). However, these findings should be interpreted with caution as stability analysis suggested that these subsample networks were not estimated with sufficient accuracy due to small sample sizes (see Additional file 1 for details).

### Community detection

Symptoms that cluster together in communities may be part of the same latent variable or dimension. The Walktrap algorithm was applied to the network to estimate the presence of symptom clusters [58]. The algorithm recursively takes random walks between pairs of nodes to define communities as densely connected parts of the networks (where random walks get trapped). Walktrap is reported to retrieve the true generating structure across a range of conditions [58–60].

### Symptom centrality and predictability

Centrality measures quantify the inter-connectedness of each node, revealing the relative importance of nodes within the network [47]. The centrality indices presented here were chosen based on correlation stability coefficients (CS): indices were considered stable if at least 50% of children could be dropped while maintaining 95% probability of a 0.7 correlation between the centrality indices based on the full sample and those derived from subsamples [53]. The case-dropping analysis (*N* bootstraps = 2000) suggested stable node strength (CS (cor = 0.7) = 0.69), expected influence (CS (cor = 0.7) = 0.75), and bridge strength CS (cor = 0.7) = 0.52). Strength represents the sum of all edge weights (regularised partial correlations) directly linked to a given node; expected influence is based on the same formula while taking negative relationships into account [61]; and bridge strength represents the sum of edge weights of given node to all nodes of a different cluster. In other words, bridge strengths highlight symptoms with multiple strong conditional relationships with symptoms from other clusters. Due to the cross-sectional nature of the data, the conceptual interpretation of these metrics is unclear. One possibility is that symptoms of high centrality may be the cause or the consequence of the symptoms they are related to. To ensure centrality indices are not biased by item properties such as differential variability of network nodes, the associations across node centrality measures and standard deviations was estimated. They were weak and non-significant (strength: *rs* = 0.23, *p* = 0.30; expected influence: *rs* = 0.22, *p* = 0.31; bridge strength: *rs* = 0.02, *p* = 0.92).

The predictability of individual symptoms was examined to quantify the shared variance between a given node and all of the symptoms connected to it (i.e. proportion of variance explained) [48]. The calculation of node predictability was based on listwise complete correlations (*N* = 668). The estimated predictability network was very similar to the network based on pairwise complete correlations (adjacency matrix correlation: *r* = .93).

## Results

### Descriptive statistics

**Fig. 1:**
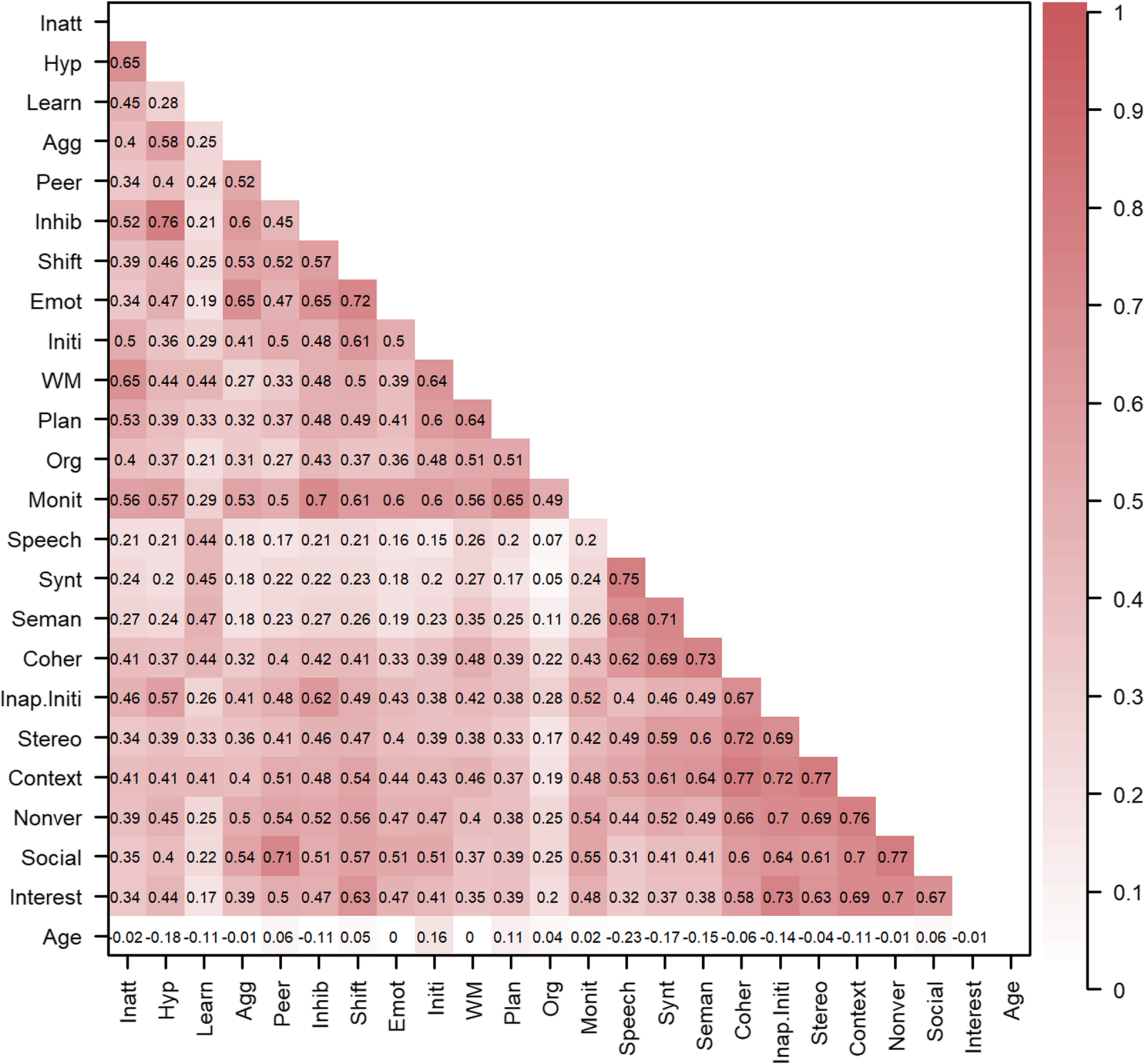
Pearson correlations after nonparanormal transformation across all variables in the network. Darker colour reflects stronger associations. Conners-3: Inatt = Inattention; Hyp = Hyperactivity/Impulsivity; Learn = Learning Problems; Agg = Aggression; Peer = Peer Relations; BRIEF (Behaviour Rating Inventory of Executive Function): Inhib = Inhibition; Shift = Shifting; Emot = Emotional control; Initi = Initiation; WM = Working memory; Plan = Planning/Organisation; Org = Organisation of Materials; Monit = Monitoring; CCC-2 (Children’s Communication Checklist): Synt = Syntax; Seman = Semantics; Coher = Coherence; Inap. Initi = Inappropriate Initiation; Stereo = Stereotyped language; Context = Use of Context; Nonver = Nonverbal Communication; Social = Social Relations; Interest = Interests

Means, SDs, and internal consistency indices (Cronbach’s alpha and McDonald’s omega) on the basis of polychoric correlations for all untransformed measures are presented in Table 2. Acceptable internal consistency was observed across all subscales. Pearson correlations across the measures are displayed in Fig. 1.

### Network estimation and stability

**Fig. 2:**
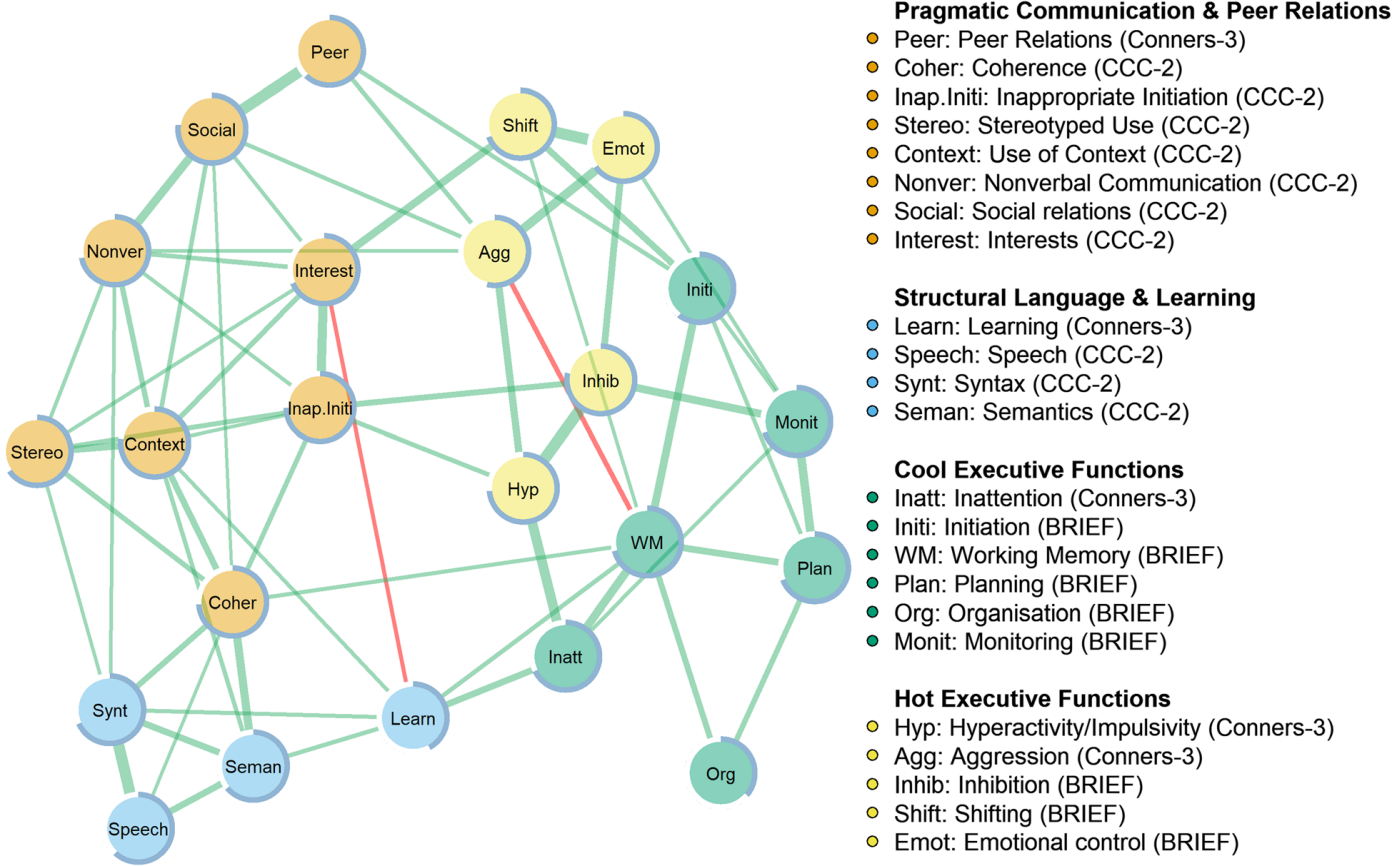
The network of behavioural and communication problems in children struggling at school. The thickness of an edge corresponds to the magnitude of the partial correlation between two nodes after adjusting for all other nodes in the network. Green edges depict positive associations and red edges depict negative associations. The node colours correspond to the clusters identified by the Walktrap algorithm. The blue ring around each node corresponds to the proportion of variance explained. BRIEF = Behaviour Rating Inventory of Executive Function; CCC-2 = Children’s Communication Checklist

The network is displayed in Fig. 2. Stability analyses indicated that the network was estimated with sufficient accuracy, as the corresponding confidence intervals were small to moderate (Additional file 1: Figure S3). All edges featured in the final network were included in the majority of the 2000 bootstrapped samples (Additional file 1: Figure S4). For edges discussed in the manuscript, the following information is included parentheses: edge weights, 95% bootstrapped confidence intervals for times the edge weight was not set to zero, and the percentage indicating how often the parameter was estimated as different from zero. For all other edges this information is available in (Additional file 1: Figure S3-S5).

### Community detection

The algorithm identified four clusters (modularity = .43) corresponding to: 1) pragmatic language and peer relationships; 2) structural language and learning; 3) cognitive skills (cool EFs); and 4) emotional and behavioural problems (hot EFs). Notably, these clusters were interrelated. Several direct paths were observed across pragmatic and behavioural difficulties (e.g. inappropriate initiation – hyperactivity (0.13, 95% CI [0.07–0.18], non-zero = 97%); inappropriate initiation – inhibition (0.19, 95% CI [0.12–0.26], non-zero = 100%). No direct relationships were observed between cool EFs and structural language; these clusters were indirectly connected through learning.

### Symptom centrality and predictability

**Fig. 3:**
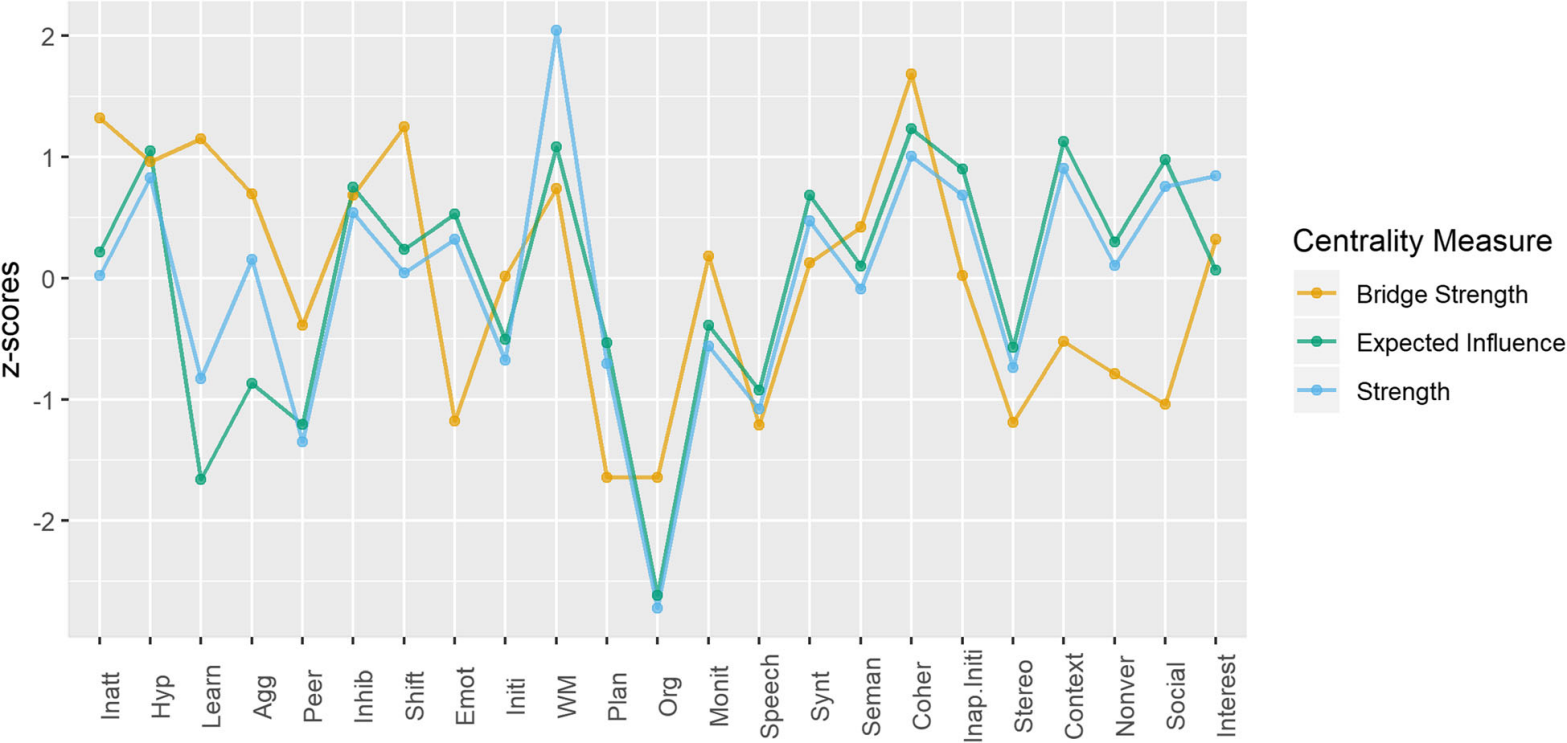
Centrality indices bridge strength, strength, and expected influence across all measures in the network. Conners-3: Inatt = Inattention; Hyp = Hyperactivity/Impulsivity; Learn = Learning Problems; Agg = Aggression; Peer = Peer Relations; BRIEF (Behaviour Rating Inventory of Executive Function): Inhib = Inhibition; Shift = Shifting; Emot = Emotional control; Initi = Initiation; WM = Working memory; Plan = Planning/Organisation; Org = Organisation of Materials; Monit = Monitoring; CCC-2 (Children’s Communication Checklist): Synt = Syntax; Seman = Semantics; Coher = Coherence; Inap. Initi = Inappropriate Initiation; Stereo = Stereotyped language; Context = Use of Context; Nonver = Nonverbal Communication; Social = Social Relations; Interest = Interests

Strength, expected influence, and bridge strength centrality are shown in Fig. 3. In the current network, working memory, coherence, use of context, and hyperactivity had the highest strength and expected influence. Coherence and shifting had the highest bridge strength (Fig. 3), reflecting multiple strong relationships with symptoms from other clusters.

Predictability analyses (Fig. 2) suggested that the symptoms explained acceptable amount of variance across the network (*M* = 0.66, *SD* = 0.10, *Min* = 0.36 (Organisation), *Max* = 0.78 (Inhibition)). A close relationship was observed between the network model and symptom predictability: if a symptom was connected to only a few other symptoms, the explained variance was lower; conversely, the more connections a symptom had, the higher the estimated predictability.

## Discussion

Network analysis was used, for the first time, to explore the co-occurrence of symptoms commonly observed across developmental disorders such as ADHD, DLD, ASD, and reading difficulties. Inter-symptom associations across communication, behavioural, and cognitive difficulties were modelled in a large heterogeneous sample of children. Four empirically-derived clusters of symptoms emerged corresponding to: 1) structural language and learning; 2) pragmatic abilities and peer relations; 3) behavioural and emotional difficulties (hot EFs); and 4) cognitive skills (cool EFs). Hot and cool EFs were directly related, as were structural and pragmatic language skills. Cool EFs were directly linked with learning but not with the formal use of language in communication.

Problems with pragmatic communication and peer relationships were directly connected to emotional and behavioural difficulties (hot EFs). In turn, behavioural difficulties were directly related to cool EFs. This was manifested in the high bridge centrality rankings of shifting and hyperactivity, both of which were in the hot EFs cluster. Their centrality in the network reflected associations with symptoms in the pragmatic communication cluster (e.g. hyperactivity – inappropriate initiation (0.13, 95% CI [0.07–0.18], non-zero = 97%); shifting – interests (0.27, 95% CI [0.22–0.34], non-zero = 100%) and the cognitive skills cluster (e.g. hyperactivity – inattention (0.36, 95% CI [0.31–0.44], non-zero = 100%); shifting – working memory (0.09, 95% CI [0.07–0.16], non-zero = 69%). These findings are consistent with hypotheses suggesting that social communication difficulties can arise as a downstream consequence of hyperactive-impulsive behaviours that are themselves underpinned by poor cool EFs [35]. The network structure cannot provide evidence for the direction of these associations, but the observed paths may imply that cool EFs (e.g. inattention, working memory) may not directly influence peer relations and pragmatic skills, but can lead to difficulties in these domains via the activation of behavioural problems (e.g. hyperactivity, shifting) [37, 62].

Contrary to expectations, structural language skills and cool EFs were not directly linked. It has been previously reported that cool EFs such as verbal short-term and working memory underpin language development [63]. However, no such links were found in the network. One possibility is that the development of structural communication skills is constrained by phonological processing, which was not included in the current study, and not by executive function [25, 64]. Links between both clusters and learning may reflect how cognitive deficits (e.g. working memory, inattention) and structural language skills (e.g. syntax, semantics) limit learning in different ways. Language impairments arising from phonological difficulties might be more closely related to literacy problems [25], with cool EF problems impacting on broader aspects of learning [32]. An alternative possibility is that cool EF deficits constrain learning, and this impairs the acquisition of structural language abilities [31]. It is further possible that the direct associations were not observed due to potential poor specificity of the regularisation methods used.

Multiple direct paths linked the structural and pragmatic communication clusters, but their associations with other symptoms in the network differed. This supports the view that these dimensions of communication are linked but could have distinct origins [65]. As suggested in the network, impairments in pragmatic language may arise through both structural language problems [66] and social/behavioural difficulties [67]. Structural language abilities appear to be more closely tied to learning, and indirectly to cool EFs.

Centrality indices highlighted important roles for working memory, language coherence, and the appropriate use of context in communication. Working memory and language coherence had multiple connections both within and outside their own clusters. Symptoms with multiple connections across problem areas may potentially interact with other areas of difficulty and may be the origin/consequence of co-occurrences [40]. Working memory bridged the cool EF and structural language clusters via learning (0.12, 95% CI [0.10–0.19], non-zero = 76%). The role of working memory in learning is well-established [68]. Working memory also linked the cool EF and pragmatic clusters via coherence (0.11, 95% CI [0.07–0.16], non-zero = 93%), and shared direct paths with hot EFs (shifting: 0.09, 95% CI [0.07–0.16], non-zero = 69%). Working memory did not rank highest on bridge strength, but its highest overall strength and expected influence, together with its direct associations with all symptoms within the cool EFs cluster and direct links with symptoms from all other clusters, provide evidence about its potential role as an area of difficulty that may spread activation across the network. Although causal conclusions on the basis of correlational network models are not warranted, working memory is important for holding information in mind, focusing attention, and ignoring distractions. This may explain how impairments activate difficulties in other problem areas such as producing coherent speech/narratives and shifting on to novel activities. Considering working memory as a transdiagnostic risk factor for developmental disorders fits with data from the mental health field [69].

Coherence in communication (pragmatic abilities and peer relations cluster) was associated with all three symptoms of structural communication (edge weights range: 0.08–0.28, non-zero range: 70–100%) and four symptoms in its own cluster (edge weights range: 0.07–0.19, non-zero range: 72–100%). Poor language coherence may therefore activate, or be the consequence of, multiple other communication problems. In contrast, the high centrality of the use of context when communicating reflected multiple links within the pragmatics and peer relations cluster (edge weights range: 0.09–0.23, non-zero range: 90–100%) and a single association with the structural cluster (semantics: 0.12, 95% CI [0.07–0.17], non-zero = 97%). Symptoms with strong connections in the same cluster may be core symptoms of this problem area [40]. Consistent with this, the appropriate use and interpretation of language in relation to context is central to the definition of pragmatics, and difficulties in this specific ability are reported to differentiate across children with primarily structural versus pragmatic difficulties [70].

### Limitations & future directions

There are several limitations to this study. First, the symptom relationships identified are based on parent ratings that are designed to capture aspects of functioning distinct from those measured in lab-based assessments of cognition [71]. Parent ratings provide ecologically valid assessments of children’s day-to-day functioning across different situations [71], but are known to be subject to reporter bias. To test whether bias affected the results, the current analyses were conducted both including and excluding reports flagged as overly negative or inconsistent. The overall pattern of results did not differ between analyses, indicating that such reporter bias is unlikely to be the cause of the overall network. In addition, the identification of clusters of symptoms that align with theoretical constructs provides further validation that the ratings were meaningful as they effectively distinguished between different aspects of functioning. It will be interesting to explore whether a similar network structure emerges with objective assessments of language, behaviour, and cognition.

The communities of symptoms identified depend on the chosen community detection algorithm and the stability of the input network. The algorithm applied here is reported to identify network communities with an acceptable level of accuracy, and the input network was stable in terms of the strength and number of connections across symptoms. Furthermore, the problem areas present in the network map on to broad latent constructs identified in previous studies of these comorbidities [3], suggesting some level of agreement across methods.

The heterogeneous nature of the sample was suited for investigating the possibility of transdiagnostic symptom-level associations across comorbid difficulties. Nonetheless, it is also possible that disorder-specific associations may have been masked by combing multiple diagnostic groups. The current study was too small in size to formally test this possibility, but coarse checks based on comparing a network for the largest diagnostic group in the sample (those with ADHD) to a network based on the full sample showed acceptable correspondence. An important direction for future research will be to compare networks both within and across diagnoses.

The network model revealed bridging symptoms, which may provide important insights about the origins of comorbidities observed on a dimensional level, and which could be interpreted as candidate targets for interventions. However, in order for such interventions to be successful the temporal order of activation in relation to connected symptoms is of key importance. The targeted difficulty should be the cause rather than the effect of other symptoms. The current analytical framework does not afford such conclusions – central symptoms may cause other symptoms or may be the consequence of those other symptoms. Furthermore, the estimated relationships may not signal interacting areas of difficulties and may instead reflect shared item content or aetiological influences that were not included in the model. To disentangle these possibilities, it is important to evaluate whether bridging symptoms play a causal role in the co-occurrence of problem domains and to test whether interventions targeting these symptoms reduce the activation of difficulties in other areas of functioning. The incorporation of neurological, genetic, and environmental factors into network models is another important step that could provide important insights about the origins of comorbidities and their potential dynamic interactions.

## Conclusion

The co-occurrence of pragmatic communication and behavioural problems was observed in a large heterogeneous sample of children with a broad range of difficulties. This suggests these comorbidities extend beyond specific diagnostic groups (e.g. ADHD, ASD). On a practical level, these findings highlight the importance of considering potential language and communication problems among children presenting with difficult behaviour, and vice versa.

Pragmatic communication skills might be indirectly influenced by cognitive skills through mediating role of behavioural regulation. In line with this, working memory and was identified as a bridging symptom, suggesting it may spread difficulties across different domains of functioning. Working memory, inattention, and structural language difficulties, were associated with learning, in line with many previous reports.

The data presented here provide one of the first large-scale applications of network modelling to symptoms associated with a range of developmental disorders. Akin to developments in psychiatry, this investigation suggests that there might be utility in shifting away from conceptualising developmental disorders as nosological entities. Transdiagnostic approaches can enable the discovery of shared liabilities and are suited for investigating the possibility that different developmental difficulties may cause one another: each problem may be the starting point for the activation of other symptoms. Using network modelling to conceptualise developmental comorbidities as arising in dynamic causal systems can provide insights into the nature of these comorbidities and may help researchers and clinicians to formulate specific hypotheses about potential causal mechanisms and intervention strategies.

## Supplementary information

**Additional file 1: Figure S1.** Correlation across the adjacency matrices derived from networks estimated with norm-referenced (nrm.), raw, and sample-centred standardised scores (std.) with (trns.) /without (non-trns.) nonparanormal transformation based on listwise (list)/pairwise (pair) correlations. The final two matrices are derived from a networks estimated without thresholding (no-thr.) and without regularisation (unrg), respectively. The network presented in the manuscript is based on sample-centred scores, applying nonparanormal transformation and thresholding, and using pairwise correlations (std.trns.pair). **Figure S2.** Centrality measures strength and expected influence across the subscales of Conners-3, BRIEF (Behaviour Rating Inventory of Executive Function), and CCC-2 (Children’s Communication Checklist-2) estimated from unregularised, regularised thresholded, and regularised non-thresholded networks. **Figure S3.** The estimated cross-symptom edge weights are represented by the red dots and the means of the bootstrapped edge weights are represented by the black dots. The corresponding bootstrap confidence intervals indicate the edge weight accuracy. **Figure S4.** The upper triangle represents estimated edge weights, where darker shades correspond to stronger edge weights. The values in the lower triangle represent how often an edge was estimated to be non-zero in the 2000 bootstraps. **Figure S5.** The estimated cross-symptom edge weights are represented by the red dots and the means of the bootstrapped edge weights are represented by the black dots. The width of the lines corresponds to 95% confidence intervals only for the times the parameter was not set to zero. The transparency of the intervals shows how often an edge was included. Lighter lines indicate that the edge was frequently set to zero. **Figure S6.** The estimated cross-symptom edge weights are represented by the red dots and the means of the bootstrapped edge weights are represented by the black dots. The corresponding bootstrap confidence intervals indicate the edge weight accuracy.

## Data Availability

The dataset generated and analysed during the current study has not been made publicly available yet as the study is still ongoing. The data will be made publically available via managed open access once the study is complete. Analysis scripts are available from the corresponding author upon request.

## Abbreviations

ADHD: Attention deficit hyperactivity disorder
ASD: Autism Spectrum Disorder
BRIEF: Behaviour Rating Inventory of Executive Function
CCC-2: Children’s Communication Checklist
DLD: Developmental Language Disorder
EF: Executive Function
