# Supplementary material for: Transdiagnostic associations across communication, cognitive, and behavioural problems in a developmentally at-risk population: a network approach
Source: BMC Pediatr. 2019 Nov 21;19:452. doi: 10.1186/s12887-019-1818-7 (PMC6873531; doi:10.1186/s12887-019-1818-7)
Supplement: Supplementary file 1 — Additional file 1: Figure S1. Correlation across the adjacency matrices derived from networks estimated with norm-referenced (nrm.), raw, and sample-centred standardised scores (std.) with (trns.) /without (non-trns.) nonparanormal transformation based on listwise (list)/pairwise (pair) correlations. The final two matrices are derived from a networks estimated without thresholding (no-thr.) and without regularisation (unrg), respectively. The network presented in the manuscript is based on sample-centred scores, applying nonparanormal transformation and thresholding, and using pairwise correlations (std.trns.pair). Figure S2. Centrality measures strength and expected influence across the subscales of Conners-3, BRIEF (Behaviour Rating Inventory of Executive Function), and CCC-2 (Children’s Communication Checklist-2) estimated from unregularised, regularised thresholded, and regularised non-thresholded networks. Figure S3. The estimated cross-symptom edge weights are represented by the red dots and the means of the bootstrapped edge weights are represented by the black dots. The corresponding bootstrap confidence intervals indicate the edge weight accuracy. Figure S4. The upper triangle represents estimated edge weights, where darker shades correspond to stronger edge weights. The values in the lower triangle represent how often an edge was estimated to be non-zero in the 2000 bootstraps. Figure S5. The estimated cross-symptom edge weights are represented by the red dots and the means of the bootstrapped edge weights are represented by the black dots. The width of the lines corresponds to 95% confidence intervals only for the times the parameter was not set to zero. The transparency of the intervals shows how often an edge was included. Lighter lines indicate that the edge was frequently set to zero. Figure S6. The estimated cross-symptom edge weights are represented by the red dots and the means of the bootstrapped edge weights are represented by the black dots. The [file 12887_2019_1818_MOESM1_ESM.docx]

Supplementary materials

# **Subscale validity checks**

The questionnaires included in this study provide indices to detect potential inconsistencies in parent ratings and/or negative response styles. The Conners-3 contains a scale to detect overly negative responses and potential bias. In the current sample, the validity scale suggested an overly negative response style for 70 responses, which may be indicative of extreme difficulties or negative bias. The validity indices of Behavioural Rating Inventory of Executive Function (BRIEF) identified two cases as inconsistent and 67 cases as having overly negative response styles. The Children’s Communication Checklist 2 (CCC-2) consistency check score identified 46 potentially inconsistent ratings. Analyses were carried out with and without the ratings flagged as highly negative or inconsistent. The estimated regularised partial correlation network obtained after omitting all potentially biased scores retained high similarity to the network estimated with them (correlation of adjacency matrices: *r* = .92). Therefore, the final analyses reported in the manuscript are based on the full sample.

# **Alternative network estimation**

The network model presented in the paper was based on all possible pairwise correlations. To evaluate the feasibility of this method we compared the extent to which symptom interrelations based on pairwise correlations are associated with those derived from the listwise correlations (*N* =668) by correlating the regularised partial correlation adjacency matrices estimated using each procedure (**Figure 1**). The network was derived using sample-centred scaled scores to circumvent normality deviations. To check the robustness of this model, estimates derived from networks based on raw scores (without scaling) and norm-referenced scores both with and without transformation were compared. Finally, we compared the adjacency matrices derived from networks estimated without thresholding and without regularisation. All estimation methods produced similar solutions as indicated by the high correlation across the derived adjacency matrices (**Figure 1**, *r* range 1 to 0.89).


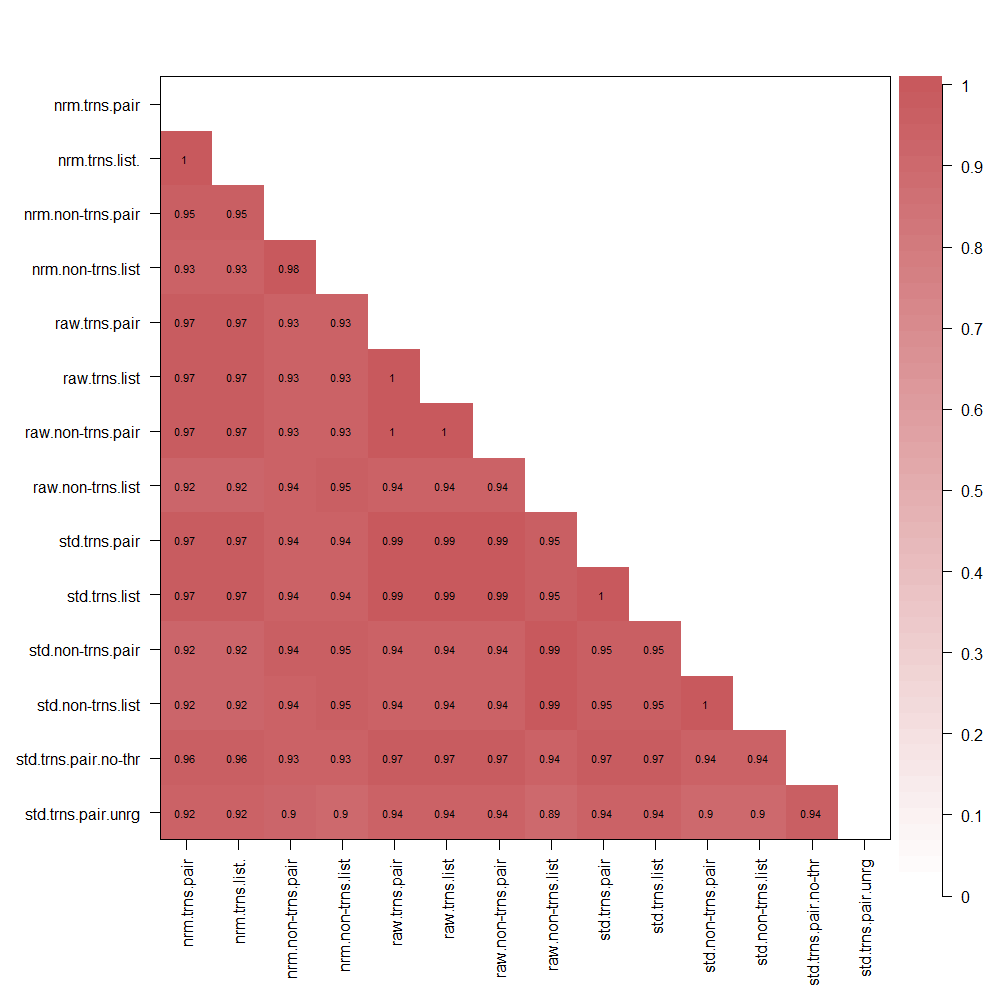


*Figure 1.* Correlation across the adjacency matrices derived from networks estimated with norm-referenced (nrm.), raw, and sample-centred standardised scores (std.) with (trns.) /without (non-trns.) nonparanormal transformation based on listwise (list)/pairwise (pair) correlations. The final two matrices are derived from a networks estimated without thresholding (no-thr.) and without regularisation (unrg), respectively. The network presented in the manuscript is based on sample-centred scores, applying nonparanormal transformation and thresholding, and using pairwise correlations (std.trns.pair).

# **Centrality measures derived from alternative estimation methods**

We scrutinised differences in centrality indices derived from alternative network estimation methods. We observed some differences across methods (**Figure 2**). Nonetheless, across all models working memory and language coherence were among the most central nodes.


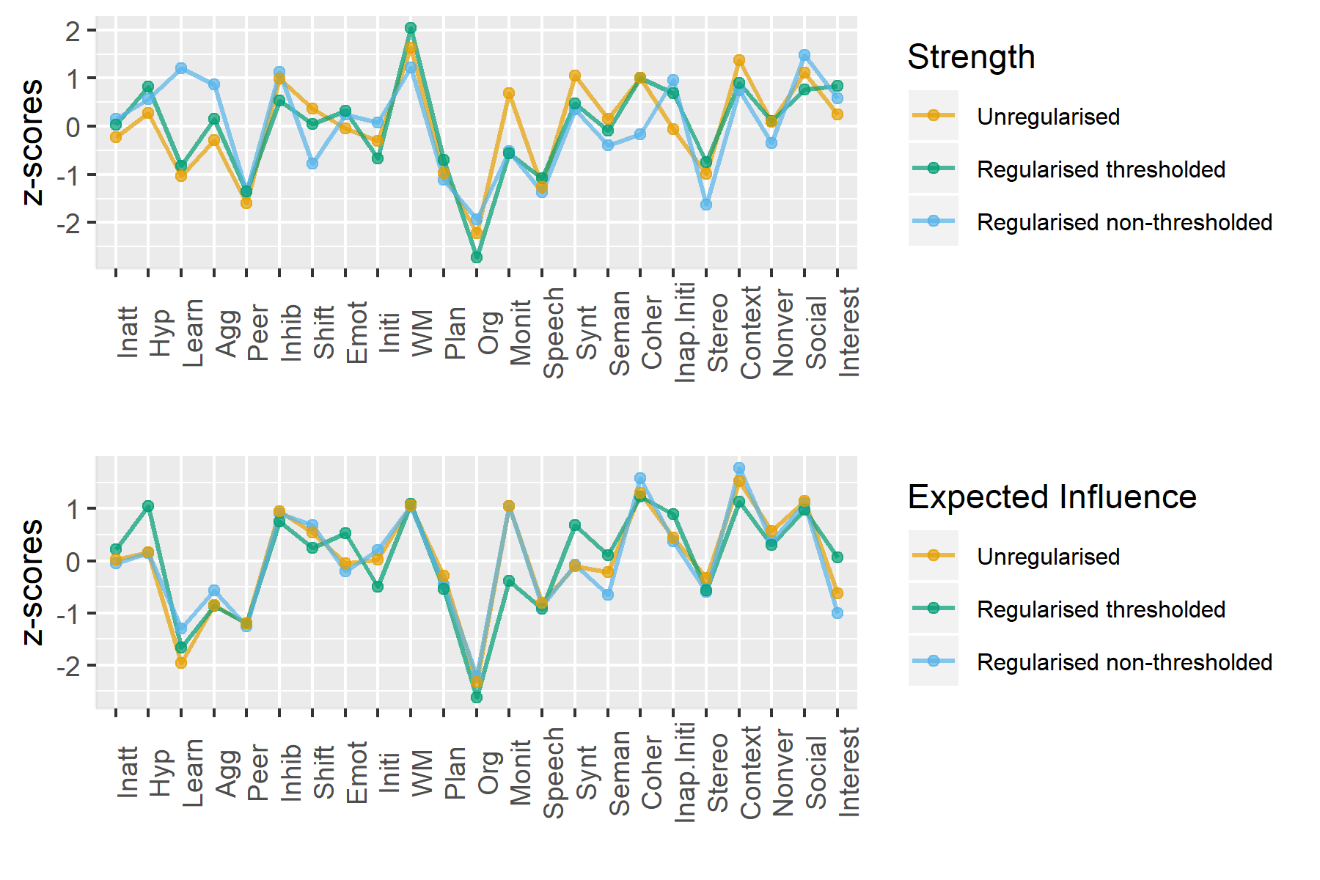


*Figure 2.* Centrality measures strength and expected influence across the subscales of Conners-3, BRIEF (Behaviour Rating Inventory of Executive Function), and CCC-2 (Children’s Communication Checklist-2) estimated from unregularised, regularised thresholded, and regularised non-thresholded networks.

*Notes.* Conners-3: Inatt = Inattention; Hyp = Hyperactivity/Impulsivity; Learn = Learning Problems; Agg = Aggression; Peer = Peer Relationships; BRIEF: Inhib = Inhibition; Shift=Shifting; Emot = Emotional Control; Initi = Initiation; WM = Working memory; Plan = Planning/Organisation; Org = Organisation of Materials; Monit = Monitoring; CCC-2: Synt = Syntax; Seman = Semantics; Coher = Coherence; Inap. Initi = Inappropriate Initiation; Stereo = Stereotyped Language; Context = Use of Context; Nonver = Nonverbal Communication; Social = Social Relations; Interest = Interests.

# **Robustness analyses**

To test the accuracy of the regularised partial correlation model the edge weights were bootstrapped (*N* boots = 2000). Edge weight confidence intervals (CIs) are useful for indicating the accuracy of edge weights and for comparing their strength. The bootstrapped CIs had acceptable widths, suggesting sufficient accuracy (**Figure 3**)*.* Notably, due to the network estimation method, the CIs are not to be interpreted as significance tests to zero^1^. Instead, sample-derived edge weights and the proportion of time edges were set as different from zero across the 2000 bootstraps are presented in **Figure 4.** The CIs derived from the times the parameter was not set to zero are presented in **Figure 5**.


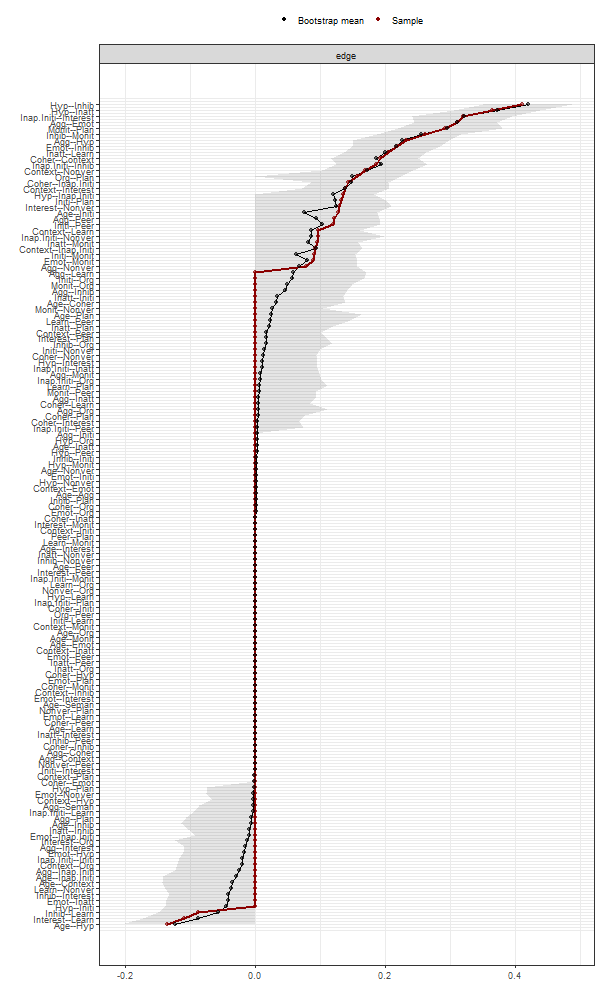


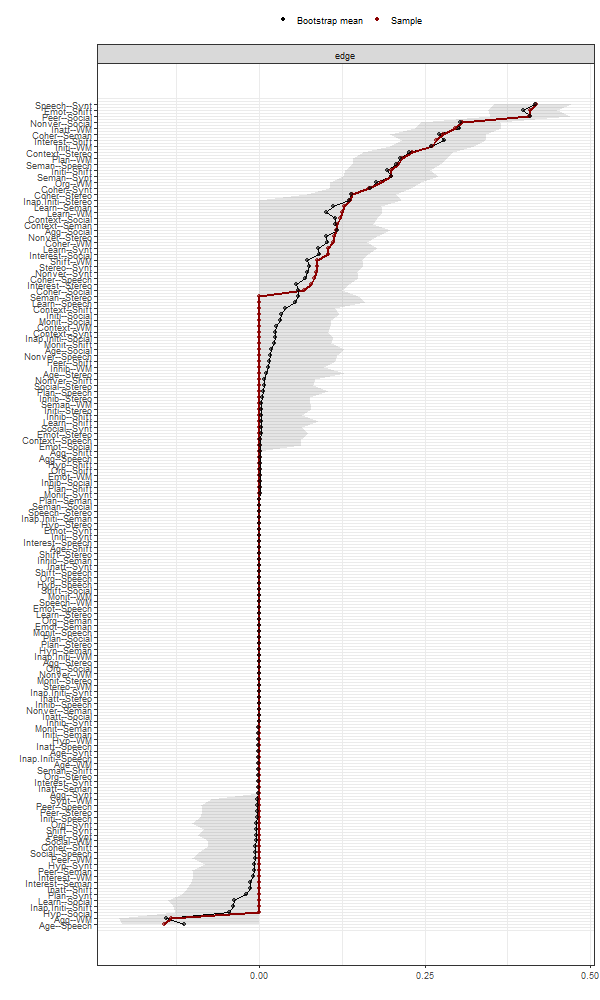


*Figure 3*. The estimated cross-symptom edge weights are represented by the red dots and the means of the bootstrapped edge weights are represented by the black dots. The corresponding bootstrap confidence intervals indicate the edge weight accuracy.

*Notes.* Conners-3: Inatt = Inattention; Hyp = Hyperactivity/Impulsivity; Learn = Learning Problems; Agg = Aggression; Peer = Peer Relationships; BRIEF: Inhib = Inhibition; Shift=Shifting; Emot = Emotional Control; Initi = Initiation; WM = Working memory; Plan = Planning/Organisation; Org = Organisation of Materials; Monit = Monitoring; CCC-2: Synt = Syntax; Seman = Semantics; Coher = Coherence; Inap. Initi = Inappropriate Initiation; Stereo = Stereotyped Language; Context = Use of Context; Nonver = Nonverbal Communication; Social = Social Relations; Interest = Interests.

**
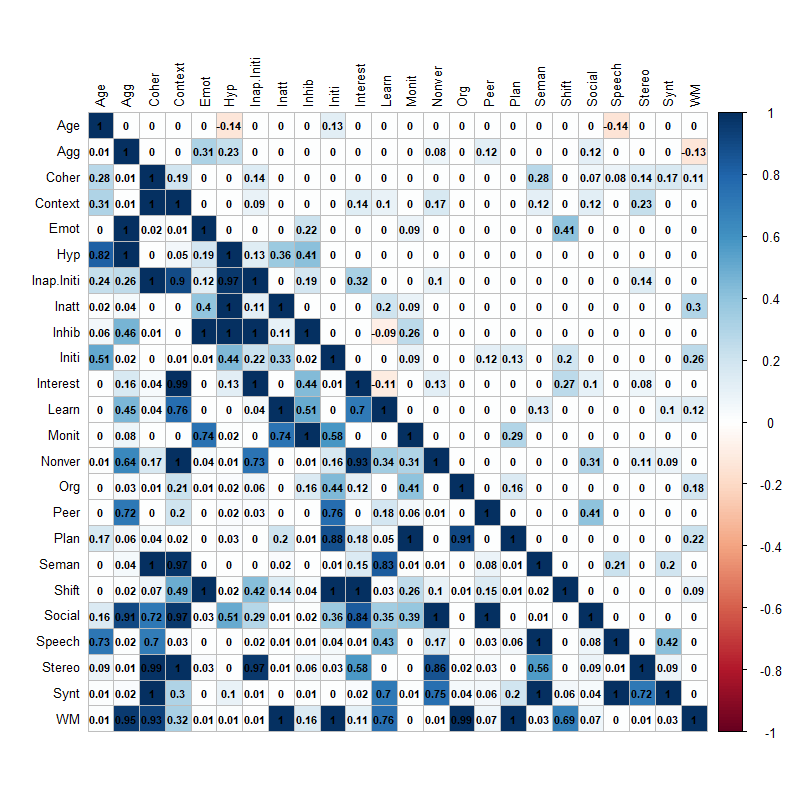
**

*Figure 4* The upper triangle represents estimated edge weights, where darker shades correspond to stronger edge weights. The values in the lower triangle represents how often an edge was estimated to be non-zero in the 2000 bootstraps. Values of 1 shaded in the darkest blue indicate that an edge was included in all networks.

*Notes.* Conners-3: Inatt = Inattention; Hyp = Hyperactivity/Impulsivity; Learn = Learning Problems; Agg = Aggression; Peer = Peer Relationships; BRIEF: Inhib = Inhibition; Shift=Shifting; Emot = Emotional Control; Initi = Initiation; WM = Working memory; Plan = Planning/Organisation; Org = Organisation of Materials; Monit = Monitoring; CCC-2: Synt = Syntax; Seman = Semantics; Coher = Coherence; Inap. Initi = Inappropriate Initiation; Stereo = Stereotyped Language; Context = Use of Context; Nonver = Nonverbal Communication; Social = Social Relations; Interest = Interests.


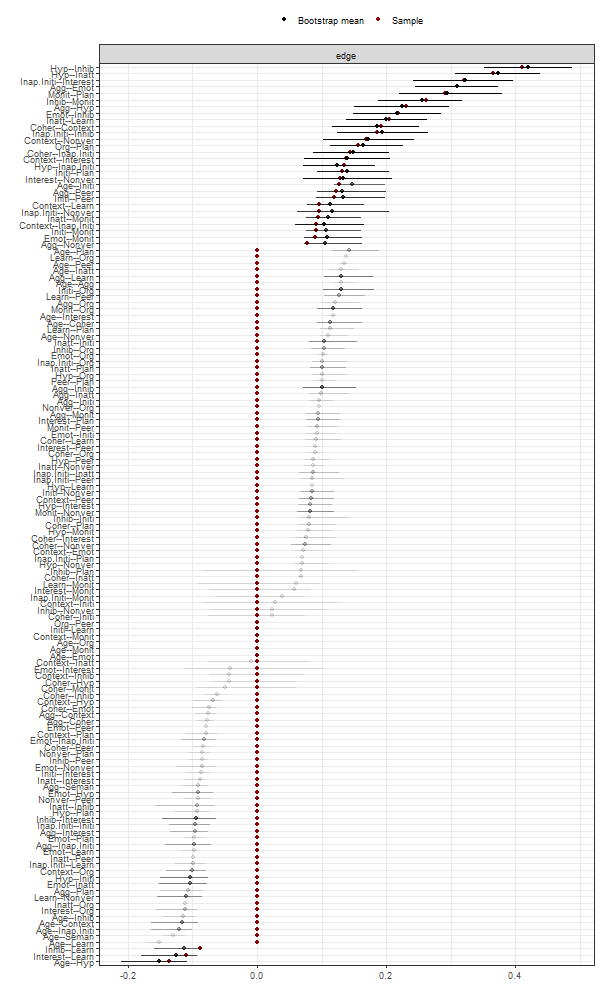


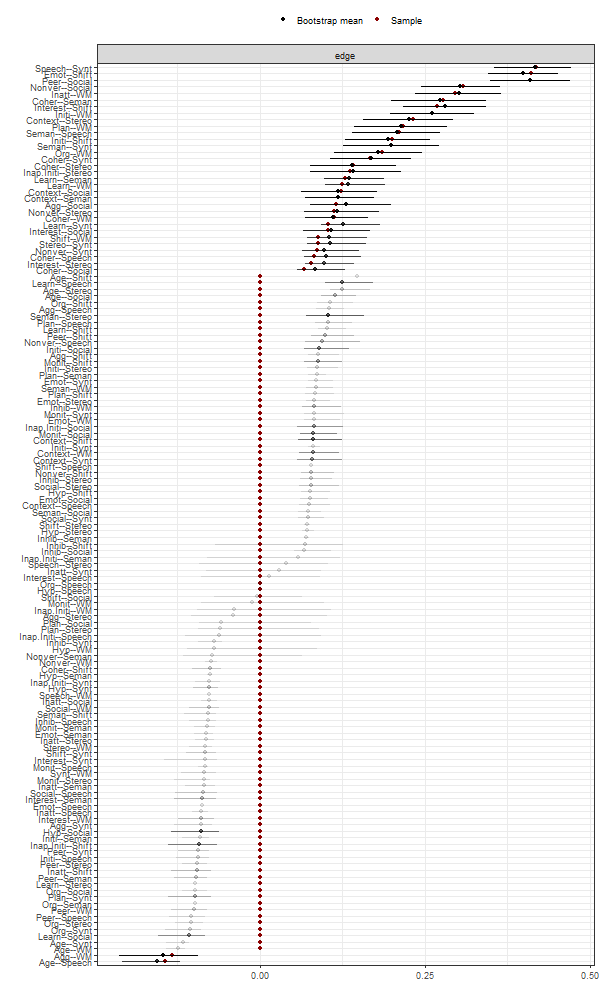


*Figure 5*. The estimated cross-symptom edge weights are represented by the red dots and the means of the bootstrapped edge weights are represented by the black dots. The width of the lines corresponds to 95% confidence intervals only for the times the parameter was not set to zero. The transparency of the intervals shows how often an edge was included. Lighter lines indicate that the edge was frequently set to zero.

*Notes.* Conners-3: Inatt = Inattention; Hyp = Hyperactivity/Impulsivity; Learn = Learning Problems; Agg = Aggression; Peer = Peer Relationships; BRIEF: Inhib = Inhibition; Shift=Shifting; Emot = Emotional Control; Initi = Initiation; WM = Working memory; Plan = Planning/Organisation; Org = Organisation of Materials; Monit = Monitoring; CCC-2: Synt = Syntax; Seman = Semantics; Coher = Coherence; Inap. Initi = Inappropriate Initiation; Stereo = Stereotyped Language; Context = Use of Context; Nonver = Nonverbal Communication; Social = Social Relations; Interest = Interests.

# **Subsample analysis: network estimation and stability**

The goal of the present study was to estimate a transdiagnostic network model. To this end, we focused on a heterogeneous sample of children with clinical and subclinical levels of difficulties. Nonetheless, it is possible that the network structure might be different across diagnostic groups. To investigate this possibility, we estimated separate networks for children with no diagnosis (*N* = 389) and children with ADHD/ADHD under investigation (*N* = 227), which was the largest represented diagnostic group. The resulting adjacency matrices were acceptably correlated *r* = .82. However, stability analysis examining the widths and overlap of confidence intervals around edge weights suggested a drop in accuracy (**Figure 6**). The stability of strength centrality was also below the recommended cut-off (ADHD/ADHD under investigation: CS (cor= 0.7) = .21; No diagnosis: CS (cor= 0.7) = .44). Given the substantial drop in accuracy we consider that the present study was underpowered to assess network differences across diagnostic groups, a possibility which should be explored in larger samples.


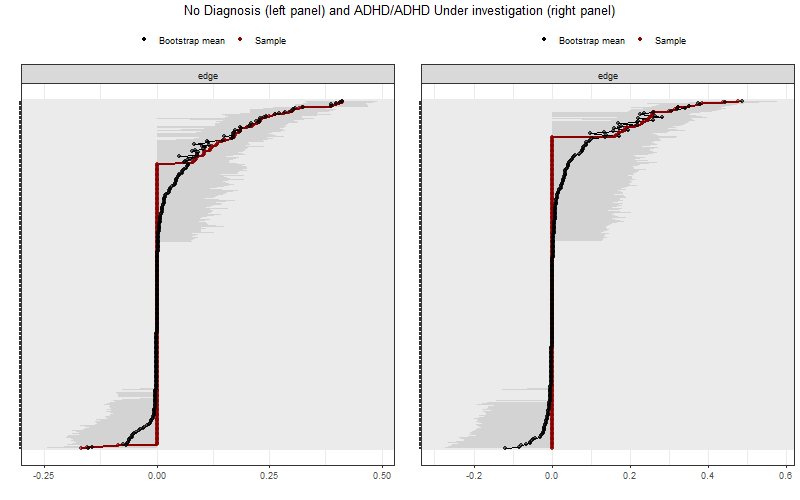


*Figure 6*. The estimated cross-symptom edge weights are represented by the red dots and the means of the bootstrapped edge weights are represented by the black dots. The corresponding bootstrap confidence intervals indicate the edge weight accuracy.

References

1 Epskamp S, Borsboom D, Fried EI. Estimating psychological networks and their accuracy: A tutorial paper. *Behav Res Methods* 2018; **50**: 195–212.
